# Safety of Nonselective Beta-Blockers in Decompensated Liver Cirrhosis and Their Role in Inducing Hepatorenal Syndrome

**DOI:** 10.7759/cureus.58296

**Published:** 2024-04-15

**Authors:** Faris Qaqish, Reem Dimachkie, Roula Sasso, Jeffrey Loeffler, Mohammed Hasan, Shabnam Deghani, Ahmad Abou Yassine, Liliane Deeb

**Affiliations:** 1 Internal Medicine, Staten Island University Hospital, Staten Island, USA; 2 Gastroenterology, Staten Island University Hospital, Staten Island, USA

**Keywords:** nonselective beta-blockers, esophageal varices, ascites, portal hypertension, acute kidney injury, liver cirrhosis, hepatorenal syndrome

## Abstract

Background

Nonselective beta-blockers (NSBBs) have been used in the management of portal hypertension and the prevention of initial and recurrent variceal bleeding in patients with liver cirrhosis. However, there is controversy regarding the use of NSBBs in patients with decompensated cirrhosis (DC) due to concerns over potential adverse effects, such as worsening of hepatic function and risk of hepatorenal syndrome (HRS). HRS is a serious complication of DC characterized by acute kidney injury (AKI) and progressive renal failure, and its development can lead to significant morbidity and mortality in this setting. Therefore, using NSBBs in patients with DC remains an area of ongoing research and debate. Our study aims to investigate the potential effect of NSBBs on HRS development.

Methodology

A retrospective chart review of 404 patients with cirrhosis was performed across all Northwell Health institutions between January 01, 2019, and December 31, 2020. An analysis was done on 516 patient encounters. Inclusion criteria included patients with an established International Classification of Diseases 10th Revision code of cirrhosis and AKI. After adjusting for clinical predictors, the Student’s t-test or Mann-Whitney U-test was used to compare variables between the two outcome groups (HRS vs. no HRS) for the continuous variables. Pearson’s chi-square test or Fisher’s exact test was used for the categorical variables to test if an association existed between the use of NSBBs at home and HRS. A two-sided p-value <0.05 was considered statistically significant. SAS 9.4 (SAS Institute Inc., Cary, NC, USA) was used for statistical analysis.

Results

The primary outcome was the development of HRS during the hospital stay. With a total of 109 visits with HRS, we had 21 (23.60%) reported HRS in the 89 visits where NSBBs were used at home before the hospitalization, while 88 (20.61%) HRS were observed in the 427 visits with no NSBB use at home. The use of NSBBs at home was not significantly associated with the development of HRS (odds ratio = 1.1, 95% confidence interval = 0.6-1.9, p = 0.7321). We also found that higher serum albumin on admission is associated with lower odds of HRS. In contrast, increased serum creatinine, bilirubin, presence of ascites, and use of pressors were associated with a higher risk of HRS.

Conclusions

Our study highlights the relevant safety of NSBB use in end-stage liver disease. Their use did not appear to increase the risk of developing HRS during hospitalization with DC. Further randomized controlled trials are warranted to shed more light on the efficacy, dose tolerance limits, and safety of NSBBs in decompensated end-stage liver disease.

## Introduction

Liver cirrhosis is a leading cause of mortality and morbidity across the world. In 2016, liver cirrhosis was reported to be the 11th leading cause of death, accounting for 2.2% of deaths in the world and the 15th main reason for morbidity, leading to 1.5% of disability-adjusted life years worldwide [1].

Portal hypertension, which develops secondary to increased intrahepatic vascular resistance, represents the driving factor in the natural history of cirrhosis and the development of clinical complications [2]. The occurrence of ascites, hepatic encephalopathy, gastrointestinal bleeding, jaundice, thrombocytopenia, and coagulopathy marks the transition from a compensated disease to the decompensated stage of cirrhosis. The prognosis of patients with decompensated liver cirrhosis (DC) is variable, with five‐year mortality ranging from 20% to 80% across studies. The overall disease trajectory is progressive, with declining health, increasing symptom burden, and frequent hospitalizations at the end of life.

Nonselective beta-blockers (NSBBs) have been the mainstay for the treatment of portal hypertension and prevention of initial and recurrent variceal bleeding in patients with liver cirrhosis since the 1980s [3]. NSBBs reduce portal flow and pulmonary hypertension by decreasing cardiac output (through β1 receptors) and causing splanchnic vasoconstriction (through β2 receptors) [4]. Other potential beneficial effects of NSSBs are their ability to reduce bacterial translocation and thus decrease the rate of infections, specifically spontaneous bacterial peritonitis (SBP) [5,6]. However, further studies are needed to validate this hypothesis.

However, there is controversy regarding the use of NSBBs in patients with DC due to concerns over potential adverse effects, such as worsening of hepatic function and risk of hepatorenal syndrome (HRS) [7]. HRS is a severe complication of DC characterized by acute kidney injury (AKI) and progressive renal failure, and its development can lead to significant morbidity and mortality in this setting. Therefore, using NSBBs in patients with DC remains an area of ongoing research and debate. Our study aims to investigate the potential effect of using NSBBs on HRS development in hospitalized patients.

## Materials and methods

Study population and design

We performed a retrospective chart review of 404 patients with cirrhosis across all Northwell Health institutions between January 01, 2019, and December 31, 2020. We analyzed data on 516 patient encounters. Using the assistance of our information technology department and the Northwell Health dataset, we identified the study population, patients with cirrhosis and AKI admitted to the hospital, using the corresponding International Classification of Disease, Tenth Revision Clinical Modification (ICD-10-CM) codes. Inclusion criteria included adult patients aged 18 years old and above, with an established ICD-10 code of cirrhosis and AKI during their hospital admission. AKI was defined as an increase in serum creatinine of 0.3 mg/dL or >50% increase in creatinine from baseline as per the Kidney Disease Improving Global Outcomes (KDIGO) criteria [8]. The following ICD-10-CM codes: K70.30, K70.31, K74.6, K74.60, K74.5, and K74.69 were included to extract patients with liver cirrhosis. We then identified patients with AKI on admission using the following ICD-10-CM codes: N17.0, N17.2, N17.8, N17.9. This study was approved by the Northwell Health Institutional Review Board. Patients who did not show evidence of cirrhosis or did not have an AKI were excluded from the study sample.

Data collection

Preliminarily, clinical variables, including patient demographics, medical and surgical history, laboratory data, and vital signs, were abstracted at the time of admission using our data warehouse. We then reviewed individual patient charts to confirm the presence of cirrhosis and that the diagnostic criteria for AKI were met. Additional data, including the etiology of cirrhosis and previous complications (esophageal varices, history of ascites, history of SBP), lab values such as serum creatinine and bilirubin, and demographic data were manually collected or verified for accuracy. We determined the presence of ascites via radiological images or documentation in the physical examination. For accuracy, we manually verified medication data, including home and inpatient drugs. We followed the patients’ hospital courses to confirm the diagnosis of HRS and determine the etiology of other forms of AKI.

Statistical analysis

Patient baseline characteristics and demographics, including age, gender, laboratory tests, medical history, medication history, and prior admissions and patient outcomes, were presented as mean ± SD for continuous variables, and frequencies and percentages for categorical variables. The Student’s t-test or Mann-Whitney U test (as appropriate) was used to compare variables between the two outcome groups (HRS vs. no HRS) for the continuous variables, and the Pearson’s chi-square test or Fisher’s exact test (as appropriate) was used for the categorical variables. Odds ratios (ORs) with corresponding 95% confidence intervals (CIs) were calculated for statistically significant variables. The diagnosis of HRS was established based on the definition of AKI above [8]. Our primary outcome was the association of NSBB use at home with HRS development during admission. The secondary outcomes included the association of other variables with HRS development, including serum creatinine, albumin, and bilirubin, and necessarily the presence or history of ascites. A two-sided p-value <0.05 was considered statistically significant. SAS 9.4 (SAS Institute Inc., Cary, NC, USA) was used for statistical analysis.

## Results

Patient demographics, baseline characteristics, and outcomes

From 701 cirrhotic patient encounters identified between January 01, 2019, and December 31, 2020, in our Northwell database, 516 encounters from 404 patients met our inclusion criteria of cirrhosis and AKI. There were 324 patients with a single visit, 57 patients with two visits, and 23 patients with three visits or more. Our study population was most commonly Caucasian (70.9%) and male (66.5%). The mean age at admission was 68 years (±12 years). The most common cause of identified cirrhosis was due to alcohol use disorder (N = 219, 42.4%), followed by hepatitis C-induced cirrhosis (N = 89, 17.2%), and nonalcoholic steatohepatitis (N = 85, 16.5%). The most common etiology of AKI was pre-renal AKI (N = 234, 45.3%), followed by HRS (N = 96, 18.6%) and acute tubular necrosis (ATN) (N = 76, 14.7%).

On admission, the mean Model for End-Stage Liver Disease (MELD)-Na score was 20 (±8). Mean albumin was 2.9 (±0.7) on admission, bilirubin 3.6 (±3.3), and creatinine was 2.5 (±1.8). The remaining vital signs and laboratory values are presented in Table 1 with their mean and standard deviation. In total, 237 (45.9%) patients developed an infection during their stay, with 26 cases of SBP. Our sample had an average length of stay (LOS) of 13 (±12) days with a mortality of 21.9%.

**Table 1:** Baseline demographics, clinical characteristics, and outcomes for cirrhotic patients hospitalized with AKI. BMI: body mass index; NASH: nonalcoholic steatohepatitis; Hep B: hepatitis B; Hep C: hepatitis C; HCC: hepatocellular carcinoma; AKI: acute kidney injury; HRS: hepatorenal syndrome; ATN: acute tubular necrosis; BP: blood pressure; ALT: alanine transaminase; AST: aspartate transaminase; Alk Phos: alkaline phosphatase; BUN: blood urea nitrogen; MELD: Model for End-Stage Liver Disease; LOS: length of stay; SBP: spontaneous bacterial peritonitis

Variables	n = 516, n (%) or mean (SD)
Baseline	
Age	67 (±12)
Male	343 (66.5%)
Female	173 (33.5%)
BMI	30.2 (±7.4)
Race	
African American	39 (7.6%)
Caucasian	366 (70.9%)
Asian	21 (4.1%)
Multiracial/Other	76 (14.7%)
Unknown	14 (2.7%)
Etiology of cirrhosis	
NASH	85 (16.5%)
Alcohol	219 (42.4%)
Hep B	20 (3.9%)
Hep C	89 (17.2%)
Other/Unidentified	65 (12.6%)
History of HCC	38 (7.4%)
Etiology of AKI	
HRS	96 (18.6%)
Pre-renal	234 (45.3%)
ATN	76 (14.7%)
Post-renal	15 (2.9%)
Cardiorenal	39 (7.6%)
Other	18 (3.5%)
Unidentified	38 (7.4%)
Vital signs and labs	
Temperature (F)	97.9 (±1.5)
Heart rate	88.5 (±20.9)
Systolic BP	123 (±24.8)
Diastolic BP	67.2 (±16.4)
Albumin	2.9 (±0.73)
Creatinine	2.5 (±1.8)
Bilirubin	3.6 (±3.3)
ALT	41.6 (±36.4)
AST	86.9 (±73.8)
Alk Phos	174.5 (±133.6)
BUN	47.6 (±35.2)
Hemoglobin	10.5 (±2.6)
INR	1.79 (±0.95)
Platelets	158.1 (±96.2)
Sodium (Na)	135 (±6)
MELD Score	19 (±7)
MELD-Na Score	20 (±8)
HbA1c	5.9 (±1.6)
In-hospital outcomes	
LOS (days)	13 (±12)
Death/Expiration	113 (21.9%)
Pressor requirement	135 (26.2%)
Mechanical ventilation	76 (14.7%)
ICU care	163 (31.6%)
Albumin infusion	129 (25%)
Octreotide use	86 (16.7%)
Midodrine use	108 (20.9%)
Development of infection	237 (45.9%)
Pneumonia	89 (17.2%)
SBP	26 (5%)

Correlation of patient characteristics with HRS vs. no HRS

When comparing the characteristics of patients from the two cohorts (HRS vs. no HRS), their baseline demographic data was noted to be similar. For patients without HRS, the most common cause of AKI was pre-renal AKI (N = 244, 59.6%), followed by ATN (N = 63, 15.5%) and cardiorenal syndrome (N = 34, 8.4%). The group that developed HRS had a statistically higher MELD-Na score, bilirubin, and international normalized ratio, and a lower albumin, platelets, hemoglobin, and sodium levels than the group without HRS. A larger proportion of patients with HRS had a history of ascites (60.6%) compared to the non-HRS group (34.6%), and most patients who developed HRS presented with ascites on admission (83.5%). Interestingly, the use of spironolactone was higher in the HRS group, possibly due to its use for underlying liver disease and ascites history. Importantly, there was a similar proportion of patients taking NSBBs at home in both cohorts, with no statistically significant difference (18.4% vs. 17%, p = 0.8417). 

Patients with HRS were more likely to have undergone a large-volume paracentesis (23.9% vs. 2.2%) and had an established SBP diagnosis (15.6% vs. 2.2%). On average, patients with HRS had a longer hospital stay (13.1 vs. 9.4 days) and a higher mortality rate (39.5% vs. 17.2%). HRS patients were more likely to need critical care and mechanical ventilation and used midodrine, albumin, and vasopressor medications more commonly than the non-HRS group (Table 2).

**Table 2:** Variables associated with the development of HRS in cirrhotic patients hospitalized with AKI. BMI: body mass index; NASH: nonalcoholic steatohepatitis; Hep B: hepatitis B; Hep C: hepatitis C; HCC: hepatocellular carcinoma; AKI: acute kidney injury; HRS: hepatorenal syndrome; ATN: acute tubular necrosis; BP: blood pressure; ALT: alanine transaminase; AST: aspartate transaminase; Alk Phos: alkaline phosphatase; BUN: blood urea nitrogen; MELD: Model for End-Stage Liver Disease; NSBB: nonselective beta-blockers; LVP: large-volume paracentesis; LOS: length of stay; SBP: spontaneous bacterial peritonitis

	+HRS (n = 109, 29.4%), n (%) or mean (SD)	-HRS (n = 407, 70.6%), n (%) or mean (SD)	P-value
Baseline			
Age	66.3 (±12.8)	68.3 (±12.5)	0.2812
Male	69 (63%)	274 (67.3%)	0.4996
Female	40 (36.7%)	133 (32.7%)	
BMI	29.9 (±7.5)	29.6 (±6.8)	0.6406
Race			
African American	4 (3.7%)	35 (8.6%)	0.1665
Caucasian	83 (76.2%)	283 (69.5%)	
Asian	5 (4.59%)	16 (3.93%)	
Multiracial/Other	12 (11%)	64 (15.7%)	
Unknown	5 (4.59%)	9 (2.21%)	
Etiology of cirrhosis			
NASH	23 (21.1%)	62 (15.2%)	0.1864
Alcohol	49 (44.9%)	170 (41.8%)	0.6252
Hep B	2 (1.83%)	18 (4.42%)	0.3352
Hep C	21 (19.3%)	68 (16.7%)	0.6275
Other/Unidentified	17 (15.6%)	76 (21.4%)	0.2296
Etiology of AKI			
Pre-renal		244 (59.6%)	
ATN		63 (15.5%)	
Post-renal		15 (3.7%)	
Cardiorenal		34 (8.4%)	
Other		18 (4.4%)	
Unidentified		33 (8.1%)	
Vital signs and labs			
Temperature (F)	97.8 (±1.4)	98 (±1.5)	0.2156
Heart rate	91 (±19.8)	88.2 (±20.1)	0.2519
Systolic BP	123.6 (±21.6)	122.6 (±26.5)	0.7497
Diastolic BP	66.3 (±14.5)	70.2 (±16.8)	0.0351
Albumin	2.64 (±0.72)	3.1 (±0.7)	<0.0001
Creatinine	2.24 (±2.1)	2.13 (±1.8)	0.4734
Bilirubin	6.29 (±3.8)	2.56 (±1.4)	<0.0001
ALT	42.3 (±28.4)	47 (±40.1)	0.5447
AST	97.4 (±60.9)	92 (±72.2)	0.7937
Alk Phos	179 (±132.5)	172 (±124.1)	0.6270
BUN	47 (±35.5)	42.8 (±27.4)	0.2096
Hemoglobin	9.97 (±2.4)	10.9 (±2.5)	0.0014
INR	1.96 (±1)	1.71 (±0.9)	0.0417
Platelets	132.8 (±92.4)	166.9 (±96.1)	0.0012
Sodium (Na)	133.6 (±5.3)	135.6 (±6)	0.0030
MELD-Na Score	26 (±8)	18 (±7)	0.1927
HbA1c	5.8 (±1.5)	6.1 (±1.4)	0.3070
Cirrhosis history			
History of ascites	66 (60.6%)	141 (34.6%)	<0.0001
Ascites on admission	91 (83.5%)	170 (41.8%)	<0.0001
Furosemide at home	57 (52.3%)	183 (45%)	0.2096
Spironolactone at home	44 (40.4%)	96 (23.6%)	0.0007
NSBB at home	20 (18.4%)	69 (17%)	0.8417
NSBB during hospitalization	16 (14.7%)	57 (14%)	0.9804
LVP before AKI	26 (23.9%)	9 (2.2%)	<0.0001
In-hospital outcomes			
LOS (days)	13.1 (±11.2)	9.4 (±9.1)	0.0006
Death/Expiration	43 (39.5%)	70 (17.2%)	<0.0001
Pressor requirement	51 (46.8%)	84 (20.6%)	<0.0001
Mechanical ventilation	26 (23.8%)	50 (12.3%)	0.0040
ICU care	45 (41.3%)	118 (29%)	0.0195
Albumin infusion	71 (65%)	58 (14.3%)	<0.0001
Octreotide use	43 (39.5%)	86 (16.6%)	<0.0001
Midodrine use	54 (49.5%)	54 (13.3%)	<0.0001
Development of infection	53 (48.6%)	184 (45.2%)	0.5003
Pneumonia	19 (17.4%)	70 (17.2%)	1.0
SBP	17 (15.6%)	9 (2.2%)	<0.0001

Odds and predictors for the development of HRS

Using the statistically significant variables from our previous analysis, we calculated the OR of developing HRS to determine the predictors associated with a higher risk. Our analysis showed that NSBB use was not associated with an increased risk of HRS (OR = 1.1, 95% CI = 0.6-1.9, p = 0.7321). Based on our results, the strongest predictors of developing HRS in our study sample, presented as OR (95% CI), were large-volume paracentesis 13.9 (6.3-30.6), SBP 8.2 (3.5-18.9), ascites on admission 7.05 (4.1-12.1), and history of ascites 2.9 (1.9-4.5). A higher bilirubin level (OR = 1.1, 95% CI = 1.06-1.15, p < 0.0001) was associated with developing HRS. A higher albumin was found to have a negative OR (0.4, 95% CI = 0.3-0.6), and lower sodium and hemoglobin were positively associated with HRS. The use of albumin, midodrine, octreotide, and vasopressors was predictive of HRS development, likely due to the inherent treatment regimen (Table 3).

**Table 3:** Odds and predictors for development of HRS in cirrhotic patients with AKI. INR: international normalized ratio; SBP: spontaneous bacterial peritonitis; NSBB: nonselective beta-blockers; HRS: hepatorenal syndrome; AKI: acute kidney injury; OR: odds ratio; CI: confidence interval

Variable	OR (CI)	P-value
Alcoholic cirrhosis	1.1 (0.74–1.72)	0.5503
History of ascites	2.88 (1.87–4.46)	<0.0001
Ascites on admission	7.05 (4.1–12.1)	<0.0001
Large-volume paracentesis	13.9 (6.3–30.6)	<0.0001
Diastolic blood pressure	0.99 (0.97–1)	0.0356
Hemoglobin	0.87 (0.8–0.94)	0.0016
Platelets	1 (0.98–1)	0.0012
INR	1.2 (0.99–1.4)	0.0598
Sodium	0.95 (0.92–0.98)	0.0034
Albumin	0.4 (0.29–0.55)	<0.0001
Creatinine	1.05 (0.1–1.2)	0.4739
Bilirubin	1.1 (1.06–1.15)	<0.0001
SBP	8.2 (3.5–18.9)	<0.0001
Albumin use	11.2 (6.9–18.2	<0.0001
Midodrine use	6.4 (4–10.2)	<0.0001
Octreotide use	5.5 (3.4–9.1)	<0.0001
NSBB use in hospital	1.1 (0.6–1.9)	0.8577
Spironolactone use at home	2.2 (1.4–3.4)	0.0006
Furosemide use at home	1.3 (0.9–2)	0.1737
NSBB use at home	1.1 (0.6–1.9)	0.7321

## Discussion

The use of NSBBs has a well-established role in the management of patients with liver cirrhosis due to their beneficial effects in decreasing the risk of primary and secondary esophageal varices [9,10], portal hypertension, and their benefits in decreasing the risk of ascites and SBP [2]. However, there is debate about whether the use of NSBBs could increase the risk of HRS in patients with DC due to conflicting evidence in the literature; where some studies outlined below, such as the Sersté et al. landmark study, have proposed decreased survival with NSBB use, particularly among patients with refractory ascites, partly due to an increase in the incidence of HRS.

HRS is a form of AKI that occurs in patients with DC. The updated definition of HRS by the International Club of Ascites in 2015 [8] is an increase in serum creatinine of 0.3 mg/dL or >50% increase in creatinine from baseline in 48 hours, which is consistent with the KDIGO criteria for defining AKI. HRS is a diagnosis of exclusion, and the diagnostic evaluation requires appropriate volume resuscitation and cessation of diuretics for 48 hours and exclusion of other causes of AKI. Failure to see an improvement in renal function following a 48-hour “albumin challenge” using 1 g/kg/day of 20-25% albumin, capped at a maximum of 100 g of albumin per day, confirms the diagnosis of HRS [11]. The pathophysiology of HRS is complex and associated with decreased renal perfusion due to an uncompensated hyperdynamic circulatory system and renal vasoconstriction. The current treatment of HRS is albumin and terlipressin or other vasopressors; the only curative treatment is liver transplantation [12,13].

The results of this study provide valuable insights into the association between NSBB use and the development of HRS during hospitalization. Interestingly, the study found that using NSBB at home was not significantly associated with the development of HRS. Among the visits where NSBB was used, 23.60% reported HRS, whereas 20.61% of visits with no NSBB use reported HRS. The OR for the association between NSBB use and HRS was 1.1, with a 95% CI of 0.6-1.9. The p-value associated with this analysis was 0.7321, indicating that the difference in HRS occurrence between NSBB users and nonusers was not statistically significant. These findings suggest that there may not be a strong causal relationship between NSBB use at home and HRS development.

These findings are supported by Hernández-Gea et al., where NSBB use in compensated cirrhosis was associated with a decreased risk of development of HRS and refractory ascites in NSBB responders [14]. Response to NSBB was defined as a reduction in the hepatic venous pressure gradient of >10% or >20%, which is also the target reduction found to have a significant impact on prophylaxis against first variceal bleeding and recurrent bleeding, respectively [10,15]. This is due to the reduction of portal hypertension, which is the driving factor for ascites, a known risk factor for the development of HRS [16].

A landmark study by Sersté et al. [17] demonstrated decreased survival in patients with refractory ascites taking NSBBs for variceal prophylaxis compared to the control non-NSBB group. The proposed pathophysiological mechanism is decreased cardiac output due to β-blockade and the resultant effects on renal hypoperfusion. Low cardiac output has been associated with an increased risk of HRS and an increased risk of non-response to NSBB therapy to reduce portal hypertension [1,18]. The potentially detrimental effects of NSBB in refractory ascites are supported by a more recent study that proposed the loss of autoregulatory cardiac output mechanisms as the culprit for developing HRS in those patients [19]. In addition, NSBBs were found to increase the risk of HRS in patients with cirrhosis who undergo paracentesis to diagnose SBP; this was attributed to the significant hemodynamic compromise associated with NSBB use in the setting of SBP [7]. However, this study only considered patients with grade C AKI in the analysis and, therefore, had a substantial overlap between AKI and HRS. They also did not follow patients to observe repeat incidence of SBP with NSBB treatment. NSBBs have been theorized to decrease the risk of bacterial translocation and development of SBP, which must be balanced against the risk of development of HRS, given that SBP is an independent risk factor for HRS [5,16]. Further randomized controlled trials need to be conducted to determine the long-term safety of NSBB use in cirrhotic patients who have had an SBP infection in the past.

Other factors may have a more significant impact on the occurrence of HRS during hospitalization, as shown in this study, such as serum albumin, bilirubin levels, the presence of ascites, and the use of vasopressors. These findings align with the known risk factors for HRS, as impaired renal function, liver dysfunction, and the presence of ascites are well-established contributors to HRS development [16]. The association of vasopressor use with HRS may indicate the presence of a more severe clinical condition or hemodynamic instability, which could further predispose patients to HRS.

Our results indicate a higher risk of HRS in patients with any degree of ascites (OR = 7.05, 95% CI = 4.1-12.1, p < 0.0001). However, the presence of ascites was an independent risk factor in the development of HRS regardless of NSBB use. The implication is that NSBBs may be beneficial in preventing the progression of portal hypertension and ascites, but their use may be less favorable in the setting of established refractory ascites, especially in NSBB nonresponders.

Higher albumin levels were associated with a decreased risk of developing HRS in our study. Albumin is a marker of the long-term synthetic function of the liver, and low albumin levels indicate a decrease in liver function. Hypoalbuminemia is associated with decreased intravascular volume secondary to increased fluid third spacing, which results in activation of the renin-angiotensin system and causes increased renal vasoconstriction and hypoperfusion, exacerbating the hemodynamic state leading to HRS [20]. In our study, hyperbilirubinemia was associated with a higher risk of developing HRS. Previous studies describe a potentially blunted response to the typical treatment of HRS with albumin and terlipressin or vasopressors in the setting of increased bilirubin [21].

These findings indicate the necessity to risk-stratify patients before determining whether NSBBs are appropriate based on patient comorbidities, including the severity of their AKI, degree of ascites, and concurrent hemodynamic status. NSBBs should be used with caution in patients at higher risk of developing HRS, especially in the setting of hypotension or concurrent SBP, as described above. More recent studies suggest the presence of a therapeutic window for NSBB treatment [22], which favored the use of NSBBs in earlier stages of cirrhosis compared to more decompensated stages. This study also highlights the emergence of carvedilol as a potential agent for the treatment of portal hypertension, showing more potent results than other NSBBs in decreasing portal hypertension, the risk of variceal bleeding, and the risk of ascites. One limitation of our study is the lack of classification of the different NSBB agents and their relative doses, which limits our ability to extrapolate this data.

Therefore, optimal monitoring and follow-up guidelines remain essential for patients receiving NSBB therapy. Cardiovascular monitoring includes regular blood pressure and heart rate assessments, along with periodic electrocardiograms to evaluate cardiac conduction and rhythm abnormalities. Monitoring exercise tolerance helps gauge treatment response. Noncardiovascular monitoring involves liver function tests to assess hepatotoxicity or dysfunction, renal function tests to detect decline or hepatorenal syndrome development, and monitoring metabolic parameters.

Due to the retrospective nature of this study, it carries certain limitations of which the authors are cognizant. Our study relies on accurate medical documentation and the application of appropriate ICD-10 codes. We relied on our IT department and used our Northwell Health data set to extract the patient charts and include them in our sample. Despite the manual chart review performed by the authors to confirm the accuracy of the diagnoses of HRS, it remains a difficult diagnosis to make, especially retrospectively, which may limit the accuracy of the results. We used data from 12 Northwell centers, which should increase the generalizability of the results despite different capabilities in different centers, including liver transplantations as a treatment modality. Additionally, the sample size and patient heterogeneity may influence the statistical power and generalizability of the findings.

## Conclusions

NSBBs are a mainstay of therapy in patients with liver cirrhosis and portal hypertension due to their valuable role in reducing the risk of esophageal varices. Despite the debate regarding NSBB use in DC and the concern for their role in inducing HRS, our results did not show any statistically significant association. Serum creatinine, albumin, bilirubin, and the presence of ascites are useful markers to predict the development of HRS in cirrhotic patients and could potentially help guide drug therapy. Further randomized controlled trials should explore NSBB efficacy, safety, and dose-tolerance limits in the setting of DC to better select patients to treat with NSBBs.
